# Analysis of riociguat and desmethyl riociguat by UPLC-MS/MS and its interaction with quercetin

**DOI:** 10.3389/fphar.2024.1470377

**Published:** 2024-09-18

**Authors:** Qingqing Li, Xiaohai Chen, Siping Zhang, Wanshu Li, Hangjuan Lin

**Affiliations:** ^1^ Department of Pharmacy, Ningbo Municipal Hospital of Traditional Chinese Medicine (TCM), Affiliated Hospital of Zhejiang Chinese Medical University, Ningbo, China; ^2^ The First Affiliated Hospital of Wenzhou Medical University, Wenzhou, China

**Keywords:** riociguat, desmethyl riociguat, quercetin, UPLC-MS/MS, drug-drug interaction

## Abstract

Riociguat, an orally soluble guanylate cyclase (sGC)-promoting drug, is mainly used in the clinical treatment of pulmonary hypertension (PH). In this study, a novel ultra-performance liquid chromatography-tandem mass spectrometry method was developed to quantify the concentrations of riociguat and its metabolite (M1) in plasma. The precision, stability, accuracy, matrix effect, and recovery of the methodology were satisfactory. Quercetin, a well-recognized compound, functions as a novel anticancer agent with the potential to alleviate symptoms of PH. Therefore, the potential interaction between quercetin and riociguat was investigated in this study. The levels of riociguat and M1 in rat plasma were measured using the method developed in this study to evaluate the interactions between riociguat and quercetin in rats. The results revealed that quercetin significantly inhibited riociguat and M1 metabolism with increased systemic exposure.

## 1 Introduction

Pulmonary hypertension (PH) is a rare and serious pathophysiological syndrome characterized by elevated blood pressure in the pulmonary arteries and their branches, causing an increased workload on the heart (Nazzareno, Marc et al., 2015). Chronic thromboembolic pulmonary hypertension (CTEPH) is a progressive PH caused by blood clots in the lungs. Pulmonary endarterectomy (PEA) is a crucial and only treatment option for CTEPH (David, 2015; Klinger, Chakinala et al., 2020; Irene, María Jesús et al., 2024); however, approximately 40% of patients with CTEPH are surgically inaccessible, and half of those treated with PEA experience residual or recurrent disease (Darren H, Bruce M et al., 2010; Eckhard, David et al., 2011; Joanna, Marion et al., 2011; John E, Li et al., 2016; David, Michael et al., 2017). These patients required other treatments, including riociguat.

Riociguat has been approved as an orally administered soluble guanylate cyclase (sGC) to treat PH and CTEPH by stimulating the NO-sGC-cGMP pathway (Marius M, Michael et al., 2012; Hossein A, Gerd et al., 2015; Nazzareno, Marc et al., 2015; Hossein-Ardeschir, Marc et al., 2016). Riociguat is rapidly absorbed after oral administration and has high absolute bioavailability (94%). The biotransformation of riociguat is mainly catalyzed by cytochrome P450 (CYP)1A1, CYP3A4, and CYP2C8 in the liver (Makowski, Rissmiller et al., 2015; Becker, Frey et al., 2016). CYP1A1 is primarily responsible for metabolite (M1) formation (Makowski, Rissmiller et al., 2015; Frey, Becker et al., 2018). The major active metabolite, M1, accounts for one-tenth to one-third of the biological activity of riociguat (Makowski, Rissmiller et al., 2015). It exhibited dose-proportional pharmacokinetics in pharmacokinetic studies and was rated as a low-risk drug in clinical studies (Frey, Becker et al., 2018). However, riociguat exposure was more variable in patients with PH than in healthy volunteers. There are different recommendations on the use of riociguat in patients with different degrees of kidney impairment, for instance, riociguat is not recommended for patients with PH and severe kidney injury, and requires dose adjustment for those with moderate kidney injury, whereas no dose adjustment is needed for mild kidney injury (Frey, Becker et al., 2018). A simple and rapid detection method should be established to aid in the clinical dose adjustment of riociguat and achieve more accurate monitoring of patient exposure to riociguat.

Quercetin, a natural flavonoid, is present in several foods and is beneficial to the human body (Huxley and Neil, 2003). Quercetin has been identified as a novel reagent for anticancer drugs that can inhibit the PD-1/PD-L1 interaction and weaken the inhibitory effect of PD-L1 on T cells (Jing, Lin et al., 2021). In another study, quercetin relieved PH in rats (Morales-Cano, Menendez et al., 2014). Patients with PH may ingest quercetin-rich medications or foods while consuming riociguat. Research has demonstrated that quercetin is a mixed-type CYP1A1 inhibitor (Schwarz, Kisselev et al., 2005). Metabolically, quercetin interacts with riociguat.

Consequently, in this study, the concentrations of riociguat and M1 in plasma were rapidly analyzed using ultra-performance liquid chromatography-mass spectrometry (UPLC-MS/MS) in the context of the aforementioned studies. The possible interactions between quercetin and riociguat in rats were further explored.

## 2 Materials and methods

### 2.1 Chemicals and reagents

**FIGURE 1:**
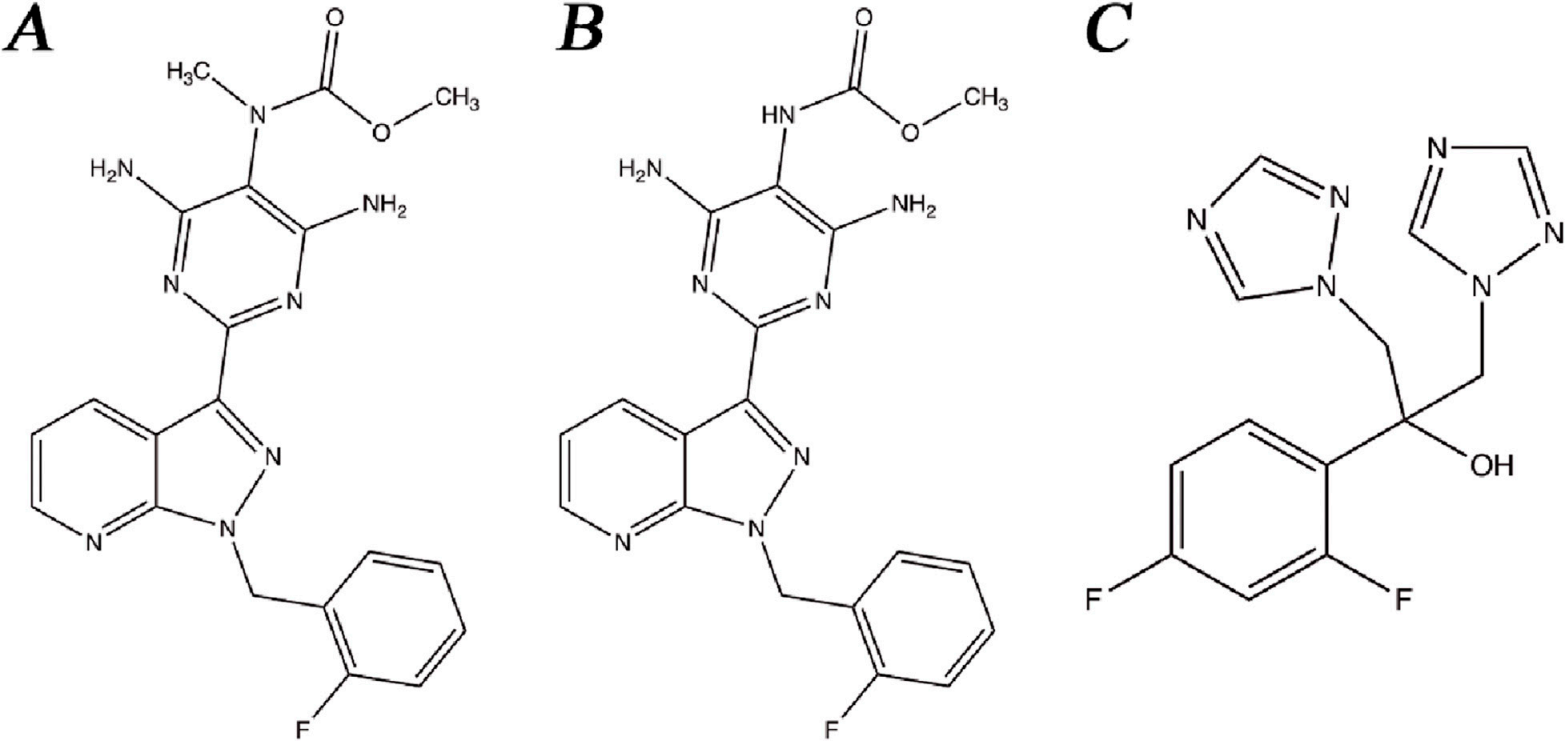
Chemical structures of riociguat **(A)**, M1 **(B)**, and fluconazole [IS, **(C)**].

Riociguat (Figure 1A), M1 (Figure 1B), and fluconazole (Figure 1C, internal standard, IS) were acquired from the Beijing Sunflower Technology Development Co., Ltd. (Beijing, China). Quercetin was obtained from Shanghai Canspec Scientific Instruments Co., Ltd. (Shanghai, China). Formic acid was bought from Anaqua Chemical Supply (Wilmington, DE, United States). Methanol and acetonitrile used in the mobile phases of UPLC-MS/MS were acquired from Merck (Darmstadt, Germany). Ultrapure water used for the instrument was supplied by a Milli-Q pure water system (Millipore, Bedford, United States).

### 2.2 UPLC-MS/MS conditions for riociguat determination and M1

**FIGURE 2:**
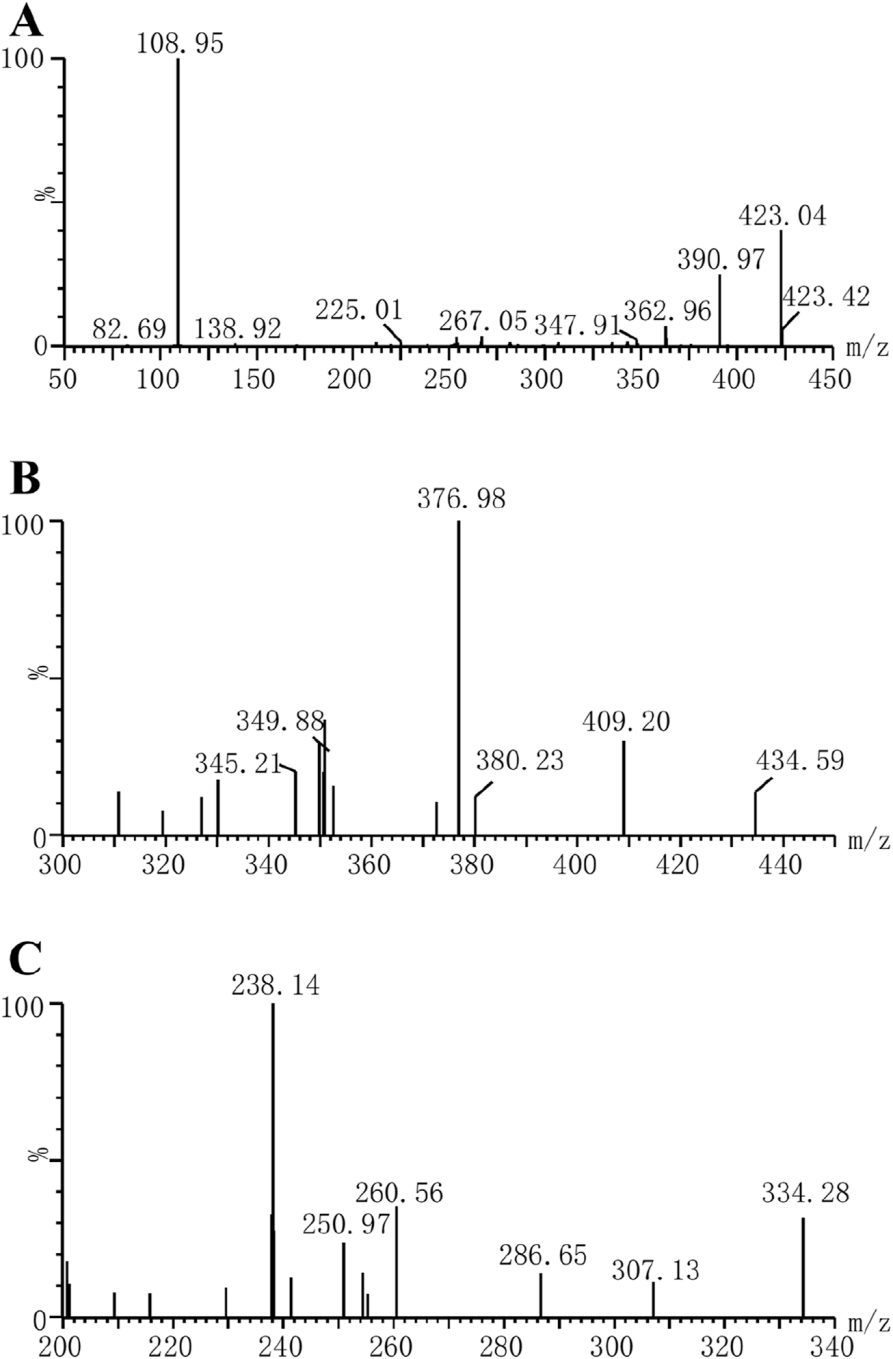
Mass spectras of riociguat **(A)**, M1 **(B)** and fluconazole [IS, **(C)**] in this study.

In this study, a Waters Acquity ultra-performance liquid chromatography system (Milford, MA, United States) equipped with an Acquity BEH C18 column (2.1 × 50 mm, 1.7 μm, Waters Corp., Millipore, Bedford, MA, United States) was used to analyze the concentrations of riociguat and M1 in plasma. The mass spectrometry parameters of the molecules were detected using a Waters XEVO TQS quadruple mass spectrometer with multiple reaction monitoring in positive mode, and the ion transitions were *m/z* 423.04 → 108.95 for riociguat, *m/z* 409.20 → 376.98 for M1 and *m/z* 307.13 → 238.14 for IS, respectively (Figure 2). Moreover, the cone voltages of the above three molecules were 30, 30, and 6 V, respectively, and the collision energies were 30, 20, and 16 eV, respectively. The mobile phase was utilized at a flow rate of 0.4 mL/min, composed of 0.1% aqueous formic acid (A) and acetonitrile (B). The elution procedure was as follows: 90% A (0.0–1.0 min), 90%–10% A (1.0–1.5 min), 10%–90% A (1.5–2.0 min), and the total running time was 2.0 min.

### 2.3 Formulation of working solutions and quality control (QC) samples

Standards, including riociguat, M1, and IS, were dissolved in methanol and separately prepared as stock solutions at concentrations of 1 mg/mL. Working solutions were prepared at a range of concentrations after gradual dilution in methanol. Different concentrations of 10 µL of riociguat (10, 20, 50, 100, 200, 500, 1,000, and 2000 ng/mL) and 10 µL of M1 (5, 10, 20, 50, 100, 200, 400, and 500 ng/mL) working solutions were mixed with 80 µL of rat plasma without drug to obtain the corresponding calibration curves.

The same procedure was used to prepare QC samples at three different concentrations (2, 80, and 160 ng/mL for riociguat and 1, 20, and 40 ng/mL for M1, respectively). All preformulated solutions were saved at −80 °C for further use.

### 2.4 Sample processing

Acetonitrile was added to prepare the samples to achieve rapid and efficient protein precipitation. In a 1.5 mL polypropylene centrifuge tube, 100 µL of the plasma sample was mixed with 20 µL of 500 ng/mL IS solution and 300 µL of acetonitrile. After thorough vortexing for 3 min, the tubes were centrifuged at 13,000 rpm for 10 min at 4 °C. The obtained supernatant (100 µL) was transferred to a new autosampler vial, and the quantitation volume was 2 µL.

### 2.5 Method validation

The analytical methods were performed following the Food and Drug Administration’s “Guidance for Industry: Bioanalytical Method Validation,” including calibration curves, the lower limit of quantification (LLOQ), precision, selectivity, stability, recovery, matrix effects (ME), and accuracy.

#### 2.5.1 Selectivity

Selectivity was evaluated by examining the presence of interference in blank plasma (from six different rats), standard solutions, and real rat plasma after dosing.

#### 2.5.2 Sensitivity and linearity

The calibration solutions of riociguat and M1 were conducted over 3 days (n = 3) and ranged from 1.0 to 200 and 0.5–50 ng/mL, respectively. The weighted (1/*x* ^*2*^) least-squares regression model was used to assess linearity by plotting the ratio of analyte peak area to IS peak area relative to the nominal analyte concentration. The LLOQ is the smallest concentration of the calibration curve required to meet certain precision and accuracy expectations (accuracy within ± 20%, precision ≤ 20%).

#### 2.5.3 Accuracy and precision

Accuracy was assessed by measuring the concentration as a percentage of the nominal concentration (relative error RE, %), and precision, indicating consistency between repeated measurements, was characterized by the relative standard deviation (RSD, %). The intra- and inter-day accuracy and precision were examined by monitoring the actual concentrations of the four QC levels (1, 2, 80, and 160 ng/mL for riociguat and 0.5, 1, 20, and 40 ng/mL for M1) on the same day or over 3 days. According to the requirements, the variation in intra-day precision and accuracy, and inter-day precision and accuracy should be less than ± 15%.

#### 2.5.4 ME and extraction recovery

The ME was calculated by comparing the response values obtained when riociguat (2, 80, and 160 ng/mL) and M1 (1, 20, and 40 ng/mL) were added to the post-extracted blank plasma with those obtained when the corresponding concentration of analytes was added to methanol (n = 5). The recovery of the preparation method was examined by comparing the ratio of response values, while riociguat (2, 80, and 160 ng/mL) and M1 (1, 20, and 40 ng/mL) were added to the blank plasma before and after extraction, respectively (n = 5). Moreover, both the RSD% of the ME and the recovery should be less than ± 15%.

#### 2.5.5 Stability

The experiments were performed at three QC levels (2, 80, and 160 ng/mL for riociguat and 1, 20, and 40 ng/mL for M1, respectively) under different storage conditions (n = 5). The conditions included stability in the analyzer (4 h, 10 °C), long-term storage (80 °C, 3 weeks), freeze-thaw cycles (three times), and short-term storage (3 h, room temperature).

### 2.6 Animal experiment

Eight Sprague-Dawley rats (200 ± 20 g) obtained from the First Affiliated Hospital of Wenzhou Medical University (Zhejiang, Wenzhou) were used in this study. During the experiment, all rats were fed a standard rodent feed and allowed unrestricted access to tap water. The animals were maintained in an environment with a temperature of 20°C–26 °C, relative humidity of 55% ± 15%, and a light-dark cycle of 12 h/day. The animal experiments were approved by the Animal Ethics Committee of the First Affiliated Hospital of Wenzhou Medical University (WYYY-IACUC-AEC-2024-037). Before the experiment, the animals were subjected to fasting for 12 h and then randomized into two groups. Quercetin (30 mg/kg) was administered to each rat in the treatment group (n = 4) by gavage (Wang, Hu et al., 2024), whereas an equal volume of 0.5% carboxymethylcellulose sodium salt was administered orally to each rat in the control group (n = 4). After 30 min, both groups were gavaged with 5 mg/kg riociguat (Lang, Kojonazarov et al., 2012; Coats et al., 2021). After riociguat treatment, blood samples were collected at 20 and 40 min and 1, 1.5, 2, 4, 6, 8, 12, 24, and 36 h. This procedure required the use of an EP tube containing heparin to collect blood samples. After centrifugation at 8,000 rpm for 5 min at 4 °C, 100 μL of plasma from the collected blood sample was added to 300 μL of acetonitrile and 20 μL of IS working solution. After centrifuging the mixture at 13,000 rpm for 10 min, the supernatant was analyzed using UPLC-MS/MS.

### 2.7 Statistical analysis

The pharmacokinetic parameters of the rats were analyzed using Drug and Statistics software (version 2.0; Shanghai University of Traditional Chinese Medicine, China). Data obtained from each experiment are displayed as mean ± standard deviation (SD) and analyzed using Statistical Package for the Social Sciences software (version 24.0). The parameters were statistically analyzed using an independent-sample *t*-test. The mean plasma concentration-time curves were plotted using GraphPad Prism software (version 9.5; GraphPad Software Inc., San Diego, CA).

## 3 Results

### 3.1 UPLC-MS/MS method development

The sensitivity of the analytes can be improved using appropriate chromatographic conditions. For the quantification of riociguat and M1, a novel UPLC-MS/MS method was developed in this analysis. Plasma samples were rapidly processed by acetonitrile protein precipitation and then separated by chromatography using a mixture of water containing 0.1% formic acid and acetonitrile. The chromatographic column chosen was a UPLC BEH C18 reversed-phase column (2.1 × 50 mm, 1.7 μm). Under suitable chromatographic conditions, favorable peak shapes and resolutions were observed for riociguat and M1.

Organic solvent precipitation refers to the promotion of the aggregation and precipitation of protein molecules through the addition of organic reagents. This is a commonly employed method for processing biological samples. The precipitation method with organic solvents is characterized by higher efficiency, convenience, and economy than methods that include liquid-liquid extraction and solid-phase extraction. After comparing various organic solvents (acetonitrile, methanol, and ethanol), this analytical method revealed that treatment of the samples with acetonitrile provided superior ME and recoveries.

### 3.2 Method validation

#### 3.2.1 Selectivity

**FIGURE 3:**
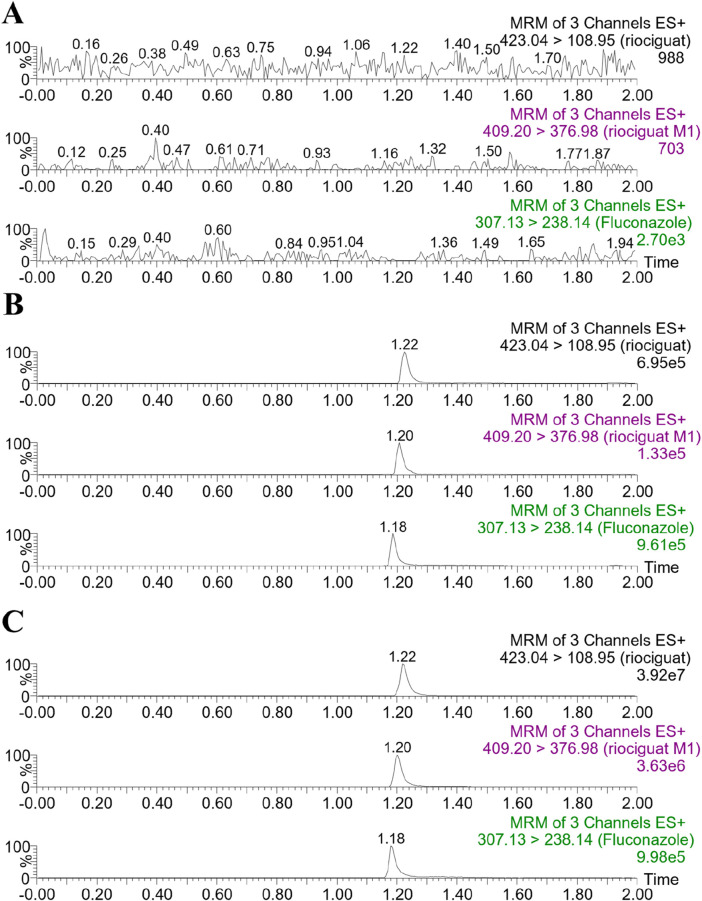
Representative chromatograms of riociguat, M1, and IS in rat plasma: **(A)** blank plasma; **(B)** blank plasma spiked with analyte at LLOQ and IS; **(C)** plasma sample collected from a rat 2 h after intragastric administration of 5 mg/kg riociguat.

The retention times of riociguat, M1, and fluconazole (IS) were 1.22, 1.20, and 1.18 min, respectively (Figure 3). The chromatograms demonstrate good selectivity and freedom from intrinsic interfering substances.

#### 3.2.2 Calibration curve and LLOQ

For riociguat, the calibration curve concentration ranged from 1.0 to 200 ng/mL, and for M1, it ranged from 0.5 to 50 ng/mL with high correlation coefficients. According to the calibration curve obtained in this study, the linear regression equation for riociguat was y = (0.259172x + 0.0880775, *r* ^*2*^ = 0.999), and M1 was represented by a linear regression equation: y = (0.133527x-0,0418774, *r* ^*2*^ = 0.999). The *r* ^*2*^ values of the two regression equations obtained were >0.99, indicating that riociguat and M1 had a satisfactory linear relationship in the corresponding ranges. The LLOQ of 1.0 ng/mL for riociguat and 0.5 ng/mL for M1 were reasonably accurate and precise.

#### 3.2.3 Accuracy and precision

The experimental process was evaluated using QC samples to determine whether the desired precision and accuracy were achieved. The concentrations of QC samples were 0.5, 1, 20, and 40 ng/mL for M1 and 1, 2, 80, and 160 ng/mL for riociguat (n = 5). According to the experimental results (Table 1), the precision and accuracy at each concentration were consistent with the standards: –15% < RE% < 15%, and RSD% < 15%. The intra-day precision ranged from 1.7% to 6.0% for riociguat, while the accuracy ranged from –0.8%–11.7%. Additionally, the inter-day precision ranged from 4.4% to 7.6%, and the accuracy ranged from –5.5%–4.3%. The intra-day precision of M1 ranged from 2.8% to 5.5%, and the accuracy ranged from –6.3%–9.6%. Furthermore, the inter-day precision ranged from 6.5% to 8.6%, and the accuracy ranged from –4.3%–1.2% (Table 1).

**TABLE 1:** Precision and accuracy of riociguat and M1 in rat plasma (n = 5).

Analytes	Concentration (ng/mL)	Intra-day		Inter-day	
		RSD %	RE %	RSD %	RE %
Riociguat	1	6.0	−0.8	7.6	−5.5
	2	1.7	8.3	4.4	3.6
	80	2.7	11.7	6.2	4.3
	160	4.5	4.0	6.0	−2.4
M1	0.5	5.5	−4.8	7.0	1.1
	1	3.7	−6.3	8.6	−4.3
	20	2.8	9.6	6.5	1.2
	40	5.2	8.4	7.3	0.1

#### 3.2.4 Recovery and ME

The formulas for calculating ME and recoveries were based on Xue’s study (Xue, Qin et al., 2019). For QC samples at 2, 80, and 160 ng/mL, the recovery of riociguat ranged from 92.3% to 99.7%, and the ME ranged from 96.4% to 113.6%. For QC samples at 1, 20, and 40 ng/mL, the recovery of M1 ranged from 97.7% to 99.9%, and the ME ranged from 99.3% to 101.5% (Table 2).

**TABLE 2:** Recovery and ME of riociguat and M1 in rat plasma (n = 5).

Analytes	Concentration (ng/mL)	Recovery (%)		Matrix effect (%)	
		Mean ± SD	RSD (%)	Mean ± SD	RSD (%)
Riociguat	2	92.3 ± 1.8	2.0	113.6 ± 5.9	5.2
	80	99.7 ± 5.8	5.8	110.7 ± 4.0	3.6
	160	93.3 ± 5.7	6.1	96.4 ± 13.1	13.6
M1	1	97.7 ± 1.0	1.0	99.3 ± 3.1	3.1
	20	98.6 ± 1.8	1.8	101.5 ± 1.8	1.8
	40	99.9 ± 1.0	1.0	100.1 ± 1.0	1.0

#### 3.2.5 Stability

The stability of riociguat and M1 in the plasma was evaluated under different treatment conditions to obtain precision and accuracy. Both riociguat and M1 exhibited good stability under different conditions (Table 3).

**TABLE 3:** Stability results of riociguat and M1 in plasma under different conditions (n = 5).

Analytes	Concentration (ng/mL)	Room temperature, 3 h		Autosampler 10°C, 4 h		Three freeze-thaw		−80°C 3 weeks	
		RSD (%)	RE (%)	RSD (%)	RE (%)	RSD (%)	RE (%)	RSD (%)	RE (%)
Riociguat	2	4.8	0.6	1.1	3.5	2.2	7.1	5.7	1.1
	80	4.7	0.9	3.7	10.3	4.8	5.0	6.1	−5.8
	160	2.9	−2.1	2.5	7.8	3.2	4.3	4.3	7.0
M1	1	2.6	−4.0	2.8	−10.8	2.1	0.8	4.5	4.5
	20	2.2	−3.9	3.3	6.4	4.1	7.6	1.8	−10.9
	40	3.5	−2.0	2.5	11.2	3.0	13.9	3.8	9.1

### 3.3 Effect of quercetin on the metabolism of riociguat and M1 *in vivo*

**FIGURE 4:**
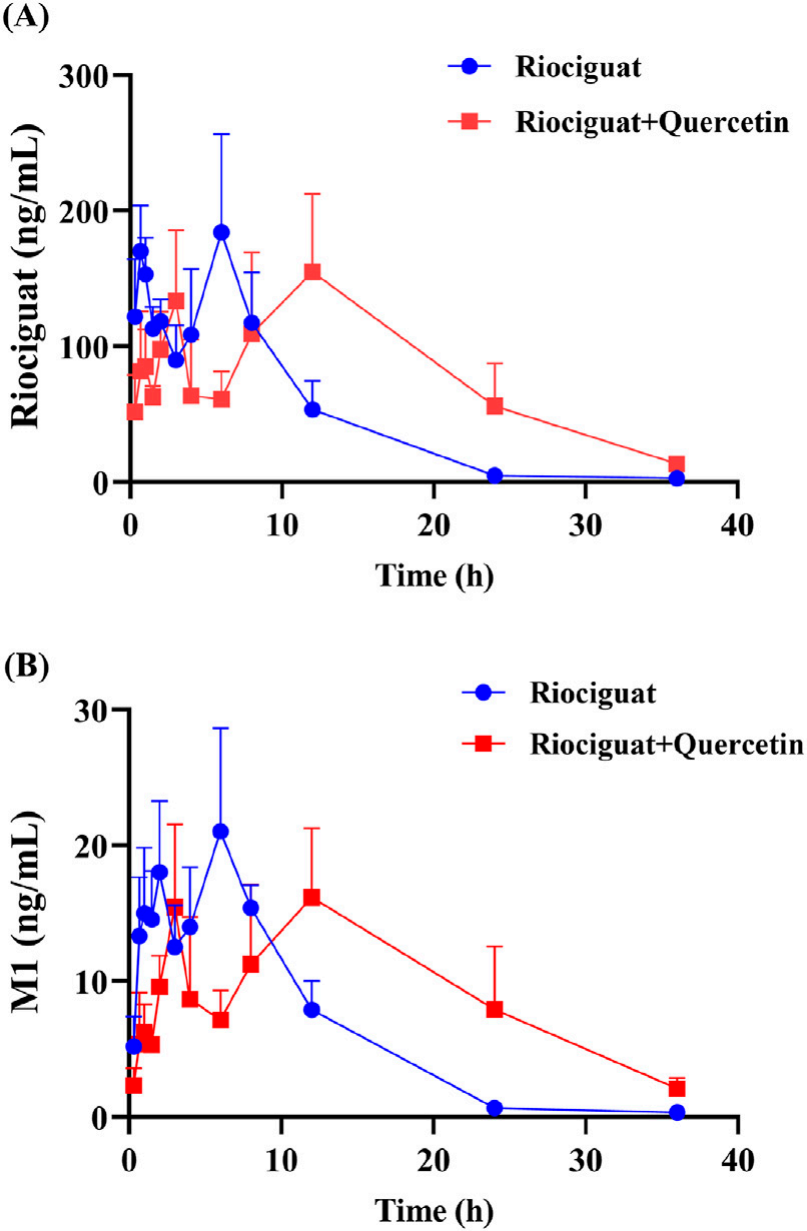
Mean plasma concentration-time curves of riociguat **(A)** and its metabolite M1 [desmethyl riociguat, **(B)**] in the control group (riociguat alone) and treatment group (riociguat with quercetin; n = 4).

The relevant parameters based on the *in vivo* pharmacokinetic experiments are displayed in Tables 4 and 5 and Figure 4. Some pharmacokinetic parameters of riociguat were significantly affected by quercetin compared to the control group, including AUC_(0-t)_, AUC_(0-∞),_ and t_1/2z_, which were increased by 61.42%, 71.85%, and 131.58%, respectively, while CLz/F was decreased by 42.86%. (Table 4). Quercetin also altered the pharmacokinetic parameters of the main active metabolite M1, with the AUC_(0-t)_, AUC_(0-∞)_, and t_1/2z_ being raised to 1.44-, 1.90-, and 3.23-fold, respectively (Table 5).

**TABLE 4:** Main pharmacokinetic parameters of riociguat in the two groups of SD rats (n = 4, mean ± SD).

Parameters	Riociguat	Riociguat + Quercetin
AUC_(0-t)_ (ng/mL^a^h)	1763.67 ± 373.10	2,846.97 ± 616.18^a^
AUC_(0-∞)_ (ng/mL^a^h)	1771.82 ± 372.94	3044.92 ± 537.01^b^
t_1/2z_ (h)	4.18 ± 0.76	9.68 ± 2.57^b^
T_max_ (h)	3.34 ± 3.08	9.75 ± 4.50
CLz/F (L/h/kg)	2.94 ± 0.74	1.68 ± 0.32^a^
C_max_ (ng/mL)	214.80 ± 50.40	156.73 ± 55.89

^a^ *p* < 0.05.

^b^ *p* < 0.01.

^c^ Compared with the Group riociguat. AUC: area under the plasma concentration-time curve; t_1/2z_: elimination half time; T_max_: peak time; CLz/F: plasma clearance; C_max_: maximum plasma concentration.

**TABLE 5:** Main pharmacokinetic parameters of M1 in the two groups of SD rats (n = 4, mean ± SD).

Parameters	Riociguat	Riociguat + Quercetin
AUC_(0-t)_ (ng/mL^a^h)	228.62 ± 36.54	328.30 ± 68.16^a^
AUC_(0-∞)_ (ng/mL^a^h)	229.38 ± 36.18	436.66 ± 107.33^a^
t_1/2z_ (h)	3.96 ± 0.58	12.78 ± 5.29^a^
T_max_ (h)	5.25 ± 2.99	9.75 ± 4.50
C_max_ (ng/mL)	23.06 ± 5.84	16.22 ± 5.20

^a^ *p* < 0.05.

^b^ *p* < 0.01.

^c^ Compared with the Group riociguat. AUC: area under the plasma concentration-time curve; t_1/2z_: elimination half time; T_max_: peak time; C_max_: maximum plasma concentration.

## 4 Discussion

Although riociguat is prescribed as a first-line drug for treating PH, its metabolism is influenced by numerous factors, including smoking, age, and hepatic and renal impairment; therefore, monitoring blood levels during clinical use is recommended (Xia, Zining et al., 2015; Becker, Frey et al., 2016; Reiner, Corina et al., 2016). Aging influences riociguat metabolism as liver and kidney functions weaken with age. When administered orally, riociguat is metabolized by cytochrome enzymes, with CYP1A1, CYP2C8, CYP2J2, and CYP3A4 accounting for approximately 27%–72% of the metabolism (Frey, Becker et al., 2018). The primary active metabolite, M1, is predominantly produced by CYP1A1 metabolism (Frey, Becker et al., 2018). Co-medication is frequent among the elderly, and before the investigation of drug-drug interactions, quantitative analysis for the simultaneous detection of riociguat and M1 is essential. Several analytical techniques have been developed in the recent years. For instance, Gnoth MJ et al. developed an LC-MS/MS method to simultaneously assay riociguat and M1 levels in human plasma (Gnoth, Hopfe et al., 2015). However, it exhibited low sensitivity, high LLOQ, and long running time. Moreover, UPLC-MS/MS was employed by Kocak OF et al. to quantify riociguat concentrations in human plasma (Kocak, Albayrak et al., 2022). Nonetheless, this analytical method failed to measure M1 concentration. In this study, we developed a novel UPLC-MS/MS method to quantify the plasma concentrations of riociguat and M1. The LLOQ, selectivity, recovery, precision, ME, accuracy, and stability of this methodology meet the required standards.

Quercetin is a class of dietary flavonoids abundantly present in several vegetables and fruits, including onions, broccoli, tomatoes, apples, strawberries, and grapes (Ko, Nile et al., 2015; Mlcek, Jurikova et al., 2016). Studies have discovered that quercetin has a certain effect on PH treatment, primarily by lowering pulmonary artery pressure (Chou et al., 2014), inhibiting right ventricular hypertrophy, and modulating vascular compliance (Morales-Cano, Menendez et al., 2014). However, the mechanism of action differed from that of riociguat. The combination of riociguat and quercetin may have a synergistic effect on the treatment of PH. Pharmacokinetic experiments in rats revealed that quercetin inhibits riociguat metabolism. Quercetin treatment prolonged the metabolic t_1/2_ of riociguat in rats, and CLz/F decreased by 42.86% (Table 4). Therefore, the mean plasma concentration-time curve of riociguat indicated that its AUC in the body was significantly increased. Schwarz D et al., quercetin is a mixed-type inhibitor of CYP1A1 (Schwarz, Kisselev et al., 2005), and M1 is mostly metabolized and produced by CYP1A1 (Frey et al., 2018). Accordingly, the outcome of the pharmacokinetic study may be attributed to the inhibition of riociguat metabolism caused by quercetin’s suppression of the CYP1A1 enzyme. Furthermore, plasma exposure to the active metabolite M1 was also increased by quercetin, with the AUC_(0-t)_ and AUC_(0-∞)_ amplified to 1.44- and 1.90-fold, respectively, and the t_1/2_ of M1 extended by 8.82 h in the group receiving riociguat and quercetin compared to the control group. This may be because M1 is primarily metabolized by uridine diphosphate glucuronosyltransferase (UGT) 1A1 and UGT1A9 (Frey et al., 2018). Quercetin is a moderate inhibitor of UGT1A1 and a strong inhibitor of UGT1A9 (Zhang, Wei et al., 2021). Overall, when riociguat was used in combination with quercetin, unpredictable adverse reactions and side effects significantly increased. In clinical practice, when patients with PH use riociguat in combination with quercetin, their plasma concentration of riociguat must be monitored in real-time.

In conclusion, riociguat and M1 metabolism in rats was inhibited by quercetin. Due to the limited number of SD rats (n = 4) in this experiment, it is necessary to repeat the tests in the future and conduct human trials for confirmation. Furthermore, its inhibitory mechanism remains unclear and needs to be verified through further experiments.

## 5 Conclusion

In this investigation, UPLC-MS/MS was used to determine the plasma concentrations of riociguat and M1. This method is fast, simple, and economical. In a pharmacokinetic study in rats, quercetin was found to have an inhibitory role in the metabolism of riociguat and M1 and increased systemic exposure. These data supported the impact of future drug interactions with riociguat.

## Data Availability

The raw data supporting the conclusions of this article will be made available by the authors, without undue reservation.
